# A188 INFORMING IMMUNOCOMPROMISED POPULATIONS: AN EFFECTIVE AND EFFICIENT COVID-19 KNOWLEDGE TRANSLATION STRATEGY

**DOI:** 10.1093/jcag/gwac036.188

**Published:** 2023-03-07

**Authors:** J W Windsor, S Coward, K Lee, A Specic, S Ghandeharian, E I Benchimol, G G Kaplan

**Affiliations:** 1 University of Calgary, Calgary; 2 Crohn's and Colitis Canada; 3 Hospital for Sick Children, Toronto, Canada

## Abstract

**Background:**

Throughout the COVID-19 pandemic, one of the major challenges was conveying expert health information, which was evolving rapidly, to confer population-level advice; this was especially relevant to at risk groups, such as those who are immunocompromised due to conditions like inflammatory bowel disease (IBD) or medications to manage disease.

**Purpose:**

To provide sufficient information to those with IBD (>0.75% of the Canadian population or roughly 300,000 individuals) and their carers to allow self assessment of personal risks related to COVID-19.

**Method:**

On March 17, 2020, Crohn’s and Colitis Canada (CCC) convened the COVID-19 & IBD Taskforce comprised of adult and pediatric gastroenterologists, IBD nurses, infectious disease experts, scientists, public health officials, communication and government relations experts, and patient advisors. The taskforce met weekly (later monthly) to synthesize rapidly evolving information on COVID-19 and personal risk assessment. Expert reviews of population-level recommendations were tailored to the IBD community and communicated through website FAQs and infographics; a public-oriented burden report with foci on additional special populations (e.g., pregnant people, pediatrics, seniors), IBD medications, and mental health and access to care during the pandemic; and through a moderated, online webinar series. The 1- to 2-hour webinar recordings were then curated into 3- to 5-minute video clips to answer specific questions and uploaded to CCC’s YouTube page. YouTube and website metrics show the continued efficacy of this strategy.

**Result(s):**

More than 24,778 households registered for the first 23 webinars, with more than one third registering for more than one webinar. As of April 1, 2021 (just after the 23^rd^ webinar), there have been 54,136 views of the archived full (1- to 2-hour) webinars and a further 78,862 views of individual webinar segments (3- to 5-minute curated clips), for a total of 126,187 views. Additionally, traffic to the CCC website increased exponentially with 484,755 unique views to the COVID-19 web pages, viewed for up to 28.29 minutes. Since April 2021, after an additional seven webinars, these numbers have continued to swell to 33,243 (registrants), 81,370 (webinar views), 92,862 (segment views), and 810,156 (unique website views); this is demonstrative of the continued impact of the electronic knowledge translation strategy.

**Image:**

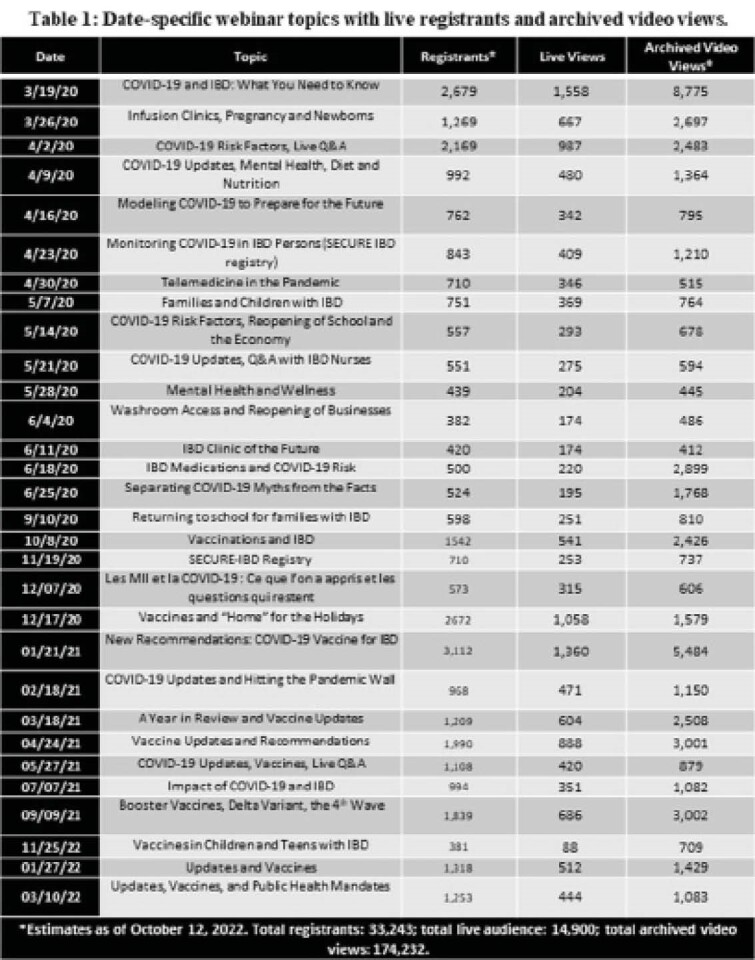

**Conclusion(s):**

While many within the IBD community were secluded during the early portions of the pandemic due to lockdown restrictions and public health advice not tailored to immunocompromised individuals, advice tailored to this community and presented through electronic methods proved to be an effective and efficient knowledge translation strategy.

**Please acknowledge all funding agencies by checking the applicable boxes below:**

CCC

**Disclosure of Interest:**

None Declared

